# Shrinking of Solid-state Nanopores by Direct Thermal Heating

**DOI:** 10.1186/1556-276X-6-372

**Published:** 2011-05-04

**Authors:** Waseem Asghar, Azhar Ilyas, Joseph Anthony Billo, Samir Muzaffar Iqbal

**Affiliations:** 1 Department of Electrical Engineering, University of Texas at Arlington, Arlington, TX 76019, USA; 2 Nanotechnology Research and Teaching Facility, University of Texas at Arlington, Arlington, TX 76019, USA; 3 Joint Graduate Committee of Bioengineering Program, University of Texas at Arlington and University of Texas Southwestern Medical Center at Dallas, University of Texas at Arlington, Arlington, TX 76019, USA

## Abstract

Solid-state nanopores have emerged as useful single-molecule sensors for DNA and proteins. A novel and simple technique for solid-state nanopore fabrication is reported here. The process involves direct thermal heating of 100 to 300 nm nanopores, made by focused ion beam (FIB) milling in free-standing membranes. Direct heating results in shrinking of the silicon dioxide nanopores. The free-standing silicon dioxide membrane is softened and adatoms diffuse to a lower surface free energy. The model predicts the dynamics of the shrinking process as validated by experiments. The method described herein, can process many samples at one time. The inbuilt stress in the oxide film is also reduced due to annealing. The surface composition of the pore walls remains the same during the shrinking process. The linear shrinkage rate gives a reproducible way to control the diameter of a pore with nanometer precision.

## Background

The use of α-hemolysin protein nanopores inspired the fabrication of solid-state nanopores. Solid-state nanopores have emerged as novel biosensors for single molecule analysis of DNA, proteins, etc. [1-7]. Solid-state nanopores are more stable than protein nanopores under various experimental conditions like pH, salinity, and temperature [8-11]. When a single bio-molecule electrophoretically passes through a nanopore, it gives significant current blockage pulses.

The diameter of the nanopore should be almost at the same scale as the size of the translocating species. The pores fabricated with conventional processes result into initial diameters larger than the size of species of interest [12-16]. The nanopore diameter is then reduced using transmission electron microscope (TEM) or field emission scanning electron microscope (FESEM) to induce the shrinking [15,17] and FIB for the sculpting processes [18]. During the TEM shrinking process, the viscous flow of SiO_2 _membrane is induced by an electron beam of optimal intensity. The nanopore shrinks or expands based on the surface-tension-driven mass flow. The nanopore, fulfilling the condition *r < t*/2, would shrink under the electron beam at optimal conditions where *r *is the radius of the pore and *t *is the thickness of the membrane. TEM beam exposure depletes oxygen from the oxide at depletion rate of about 10% per hour [15]. Higher shrinking rates can be achieved through FESEM induced shrinking [17].

The FESEM induced shrinking mechanism is putatively not surface tension driven, but explained by radiolysis. The crystalline structure of the nanopore is disturbed under a high energy FESEM electron beam. This results in pore shrinkage due to the diffusion of Si and oxygen atoms toward the edge of the pore to overcome the crystalline defects present at the edge. The stoicheometry of the SiO_2 _is expected to be different than a normal oxide layer due to radiolysis. Different shrinking rates were reported by using different acceleration voltages during FESEM exposure [17]. The nanopore was found to be always shrinking independent of the ratio of the pore's diameter and membrane thickness under FESEM [17].

During the FIB sculpting process, the nanopore is exposed to an energetic ion beam. The accelerating ions drilled a nanopore in a thin oxide membrane due to sputtering of the surface, or these reduced the pore diameter due to atom diffusion or surface tension driven mass flow [18,19]. The FIB sculpting process is also dependent on the substrate temperature. Under an Argon ion beam, the pore closed at room temperature while it opened at temperatures close to 0°C [18].

Chemical composition of the material around the nanopore periphery changes during TEM or FESEM induced shrinking processes. This produces variable modifications of nanopore surface properties. These processes make the nanopore unfavorable for molecule analysis due to increased surface charge and electrical noise in the desired signal. In addition, all these shrinking processes are time consuming because they can only process one nanopore at a time. In this article, we report a simple and novel method to shrink nanopores using direct thermal heating. High temperature treatment (>1000°C), or annealing, promotes the viscous flow of the silicon dioxide (SiO_2_) membrane and results in morphological changes that depend on the ratio of nanopore diameter to membrane thickness. Residual stress in the SiO_2 _membrane is also reduced during high temperature annealing. Surface composition of the nanopore is maintained in this approach, as opposed to being inevitably changed in the electron or ion irradiation approaches previously reported. Annealing has been extensively used in semiconductor industry to reduce leakage current in thin films [20], to repair gate oxide damage from electrical stress [21], and to minimize residual stress in amorphous films [22].

## Results and discussion

**Figure 1:**
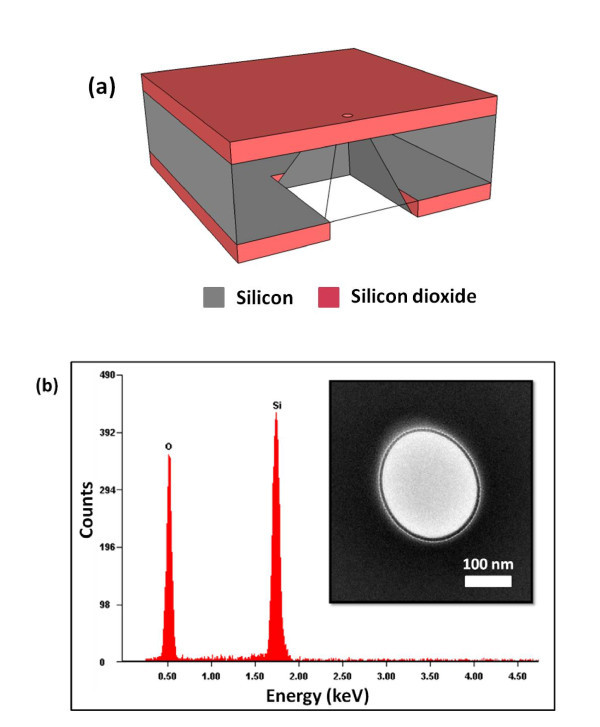
**Pore fabrication process and EDS analysis of membrane**. (a) The schematic showing a cross-section view of the device. It consists of 250 nm thick free-standing SiO_2 _membrane, in 200 μm thick wafer. The small circle on the top of the membrane depicts a nanopore drilled using FIB. (b) EDS spectrum from SiO_2 _membrane confirming the presence of only Si and O. TEM micrograph (inset) shows the nanopore in free-standing membrane drilled with FIB.

A boron-doped double-side-polished Si (100) wafer was thermally oxidized to a thickness of 400 nm. Square etch-start windows were opened in the SiO_2 _using standard photolithography. Free-standing SiO_2 _membranes (30 × 30 μm^2^) were achieved using wet tetramethylammonium hydroxide (TMAH) anisotropic etching through the whole wafer thickness. The schematic in Figure 1(a) depicts the membrane formed after anisotropic etching. Bulk membrane composition was determined by energy dispersive X-ray spectroscopy (EDS). The EDS analysis showed that the membranes contained only Si and O, as shown in Figure 1(b). The EDS analysis revealed 31% Si and 69% O. This was in good agreement with the expected stoichiometric film ratio of 33.33% Si and 66.66% O in SiO_2_. A FIB was then employed to drill nanopores in free-standing SiO_2 _membranes operated at a 30 kV acceleration voltage [23]. A larger portion of the drilled nanopores were in the diameter range of 100 to 300 nm. The high-resolution transmission electron microscope operating at 300 kV was used to image the nanopores after FIB drilling as shown in the inset of Figure 1(b). The nanopore dyes were kept in heating furnace at specific temperature for pore shrinking. The nitrogen flow rate of 20 sccm was maintained during this process.

**Figure 2:**
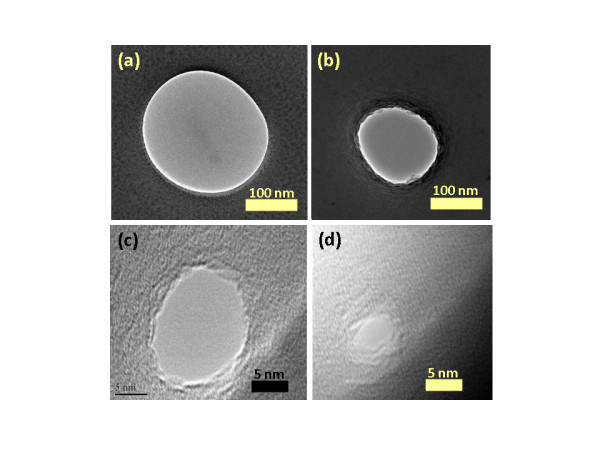
**TEM micrographs showing thermal shrinking of silicon dioxide nanopore**. (a) TEM micrograph of ~250 nm pore drilled with FIB in 300 nm thick oxide membrane. (b) TEM micrograph of the nanopore after 5 min of thermal shrinking at 1150°C. The diameter of the nanopore was ~150 nm. The wavy surface of the oxide at nanopore edges shows the shrinking process due to viscous flow of oxide. (c) Nanopore after 10 min. The diameter is ~20 nm. (d) Nanopore after another 10 min and 40 s showing the diameter of ~3 nm.

**Figure 3:**
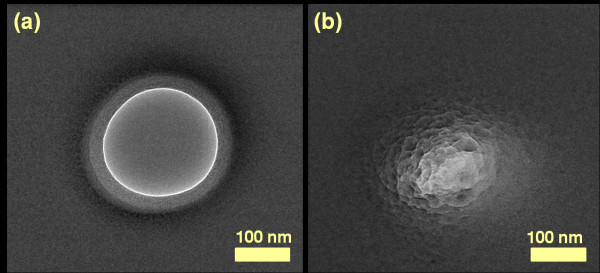
**High temperature shrinking process**. (a) TEM micrograph of ~270 nm diameter nanopore before shrinking. (b) TEM micrograph of nanopore after 4 min of thermal shrinking at 1250°C. The pore closed in just 4 min due to high shrinkage rate. The shrinking rate was about 70 nm/min.

**Figure 4:**
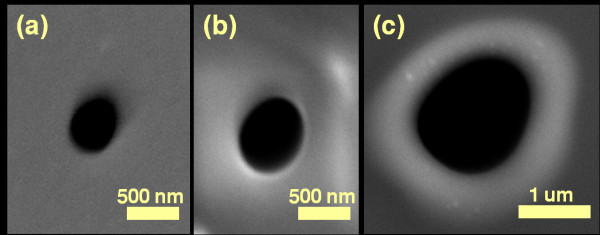
**SEM micrographs show pore expansion**. (a) The nanopore before thermal process. The initial diameter of nanopore was ~350 nm and thickness of the oxide membrane was ~300 nm. (b) The nanopore after processing at 1150°C for 15 min. The diameter increased to 650 nm. (c) The nanopore further expanded to 1.5 μm after 50 min heating at 1150°C.

We observed the nanopores shrinking or expanding when subjected to high temperature (1000 to 1250°C), contradicting previous findings [15]. The nanopores having an initial diameter of 250 nm were reduced to 3 nm at 1150°C as shown in Figure 2. The nanopores were imaged with TEM after each temperature processing step to characterize the process. After loading the dyes into the furnace, the temperature was allowed to stabilize for 30 s before counting the actual processing time. After the thermal process, the dyes were unloaded from furnace and cooled down to room temperature. When the dyes were processed at temperatures below 1000°C, it was observed that there was very little or no change in the diameter of the nanopore. This can be explained by the fact that at low temperature (<1000°C), the oxide layer would not be relaxed to an extent that it would start changing pore morphology. When the nanopores were processed at a higher temperature (>1250°C), the oxide membranes either broke due to very high thermal stress or the shrinking process was too fast to control. This was especially so for pores smaller than 20 nm diameter [24]. As an example, a nanopore with initial diameter of ~270 nm, processed at 1250°C, is shown in Figure 3. The TEM images of the nanopore show that the nanopore closed after 4 min due to an increased shrinking rate. The shrinking or expansion rate thus increased at higher temperature. When the pore diameter was larger than the membrane thickness, the nanopore started expanding in size instead of shrinking. A 350 nm nanopore in a 300 nm thick membrane was processed at 1150°C for 50 min. The pore expanded in size to 1.5 μm (Figure 4). It is interesting to note that direct heating can be used to shrink or expand the pore based only on the ratio of initial nanopore diameter to cylindrical length of the pore. The temperature itself had no effect on whether the nanopore would shrink or expand. The pore shrinking and expanding mechanism can be explained by the surface tension which induced viscous flow of oxide film as described below.

**Figure 5:**
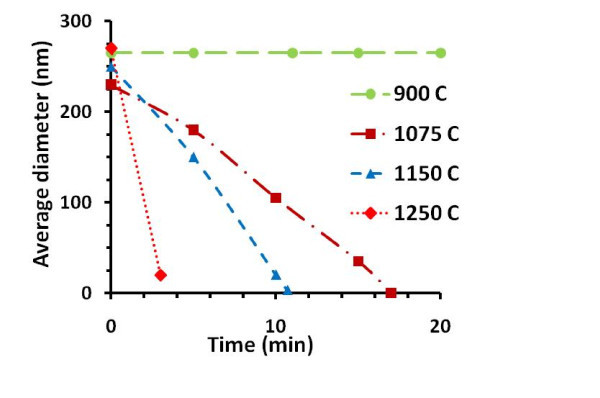
**Plot of pore diameter vs time at different temperatures**. This plot is based on TEM micrographs of different nanopores processed at different temperatures. No change in pore size is seen after 20 min at 900°C. The pore shrinkage rate increased with increasing temperature. Note: average diameter of the nanopore = sqrt (long axis × short axis). All the shrinking processes show almost linear shrinking behavior.

The nanopore shrinking process was characterized at different temperatures as shown in Figure 5. The nanopore had no shrinking or expansion at 900°C. When the temperature was increased above 1000°C, the pore morphology started changing due to the diffusion and the viscous flow of oxide. The average nanopore shrinking rate was ~22 nm/min at 1150°C, which increased to 80 nm/min when the temperature was raised to 1250°C. At higher temperatures, the shrinking process was difficult to control precisely at the nano scale. When the nanopore diameter was reduced to tens of nanometers, low processing temperature (<1150°C) was used to accurately control the shrinking.

An obvious concern in the pore shrinking process is the possibility of hydrocarbon contamination that can affect pore shrinkage dynamics. All the dyes were cleaned with Piranha solution (1:1, sulfuric acid:hydrogen peroxide) before nanopore drilling with FIB. The chips were cleaned with argon-oxygen plasma for 5 min before and after each shrinking step. The chips were also cleaned with piranha solution for 5 min after TEM images to see whether the cleaning had any effect on the nanopore size. TEM images after cleaning revealed that the pore diameter remained the same. The local EDS analysis after each processing step showed no traces of hydrocarbons as shown in Table 1. Secondly, the nanopore shrank (Figure 2) or expanded (Figure 4) based on the ratio of nanopore radius to oxide membrane thickness, which is a strong indication that hydrocarbon contamination is not involved in the shrinking process. Thus, the process is not associated with hydrocarbon contamination.

**Table 1:** EDS analysis of pore at different steps of the process.

Processing condition	Element	Wt%	At.%
Before FIB drilling	O	55.97	69.06
	Si	44.03	30.94
After FIB drilling	O	55.60	68.73
	Si	44.40	31.27
After heating	O	56.33	69.37
	Si	43.67	30.63

The analysis showed no O depletion during the process. The weight and atomic percentages of the nanopore edges remained almost constant. No traces of hydrocarbons were found at any step.

The physics of nanopore shrinkage and expansion can be explained by taking into account the surface tension of the viscous oxide membrane [15]. At high temperature, the oxide membrane softens and deforms to find a structural morphology with lower surface free energy *F*. For simplicity, the nanopore is considered cylindrical with radius *r *and oxide membrane thickness *t*. The change in free energy with respect to radius can be calculated using the simple mathematical relation Δ*F *= γΔ*A *= 2πγ (*rt *- *r*^2^), where γ is the surface tension of the fluid and Δ*A *is the change in the surface area [15,25]. From the above relation, it can be concluded that surface free energy of the nanopore having *r *<*t*/2 can be lowered by reducing *r*, whereas for nanopores having *r *>*t*/2, their surface free energy can be lowered by increasing size [15,25]. The ratio of radius to membrane thickness along with the exact geometry of the nanopore, are considered important factors in estimating a decision on whether the pore will shrink or expand. The decisive ratio of nanopore radius and membrane thickness was also verified experimentally. A 250 nm diameter pore in a 300 nm membrane shrank (Figure 2), while a 350 nm diameter pore in 300 nm membrane expanded (Figure 4) at 1150°C. Experiments performed on 150 nm thick membranes also showed similar results (data not shown). Interestingly, nanopore shrinking similar to TEM shrinking can be achieved at high temperature. The major advantage provided is that TEM processes one pore at a time, whereas this approach can process a whole wafer in one run. We believe that viscous flow is induced in the oxide membranes which results in nanopores shrinking or expanding. Similar dynamics of pore closing and opening have been reported in films of mercury and air holes in water sheets [26]. The holes used in these studies were of micrometer scale. The larger holes increased in size while the smaller holes closed down due to surface tension [26]. Similar kinetics have also been observed when 20 nm thick gold sheets with 10 to 30 nm pores were subjected to an annealing process [25]. Mathematical modeling and experiments proved that pores with diameters smaller than the gold film thickness tend to shrink while pores with diameters larger than the film thickness tend to expand during the thermal annealing process [25]. Similar diffusion kinetics of oxide membranes to shrink or expand the nanopores during high temperature annealing process may be applicable.

## Methods

### Nanopore fabrication and characterization process

The fabrication process started by oxidizing a double-side-polished, boron-doped silicon (100) wafer. The initial oxide thickness was 400 nm. Positive photoresist (PR) S1813 (Shipley Microposit J2 PR, Marlborough, MA, USA) was coated on one side of the wafer and square windows were opened after development. PR was coated on the other side followed by buffered hydrofluoric acid wet etching to remove oxide from square windows. The wafer was then washed with de-ionized (DI) water and dried with nitrogen. The wafer was submerged in acetone to remove the remaining PR. In order to make free-standing membranes, anisotropic etching was performed using 20% TMAH in DI water at 90°C (Mallinckrodt Baker, Inc. Phllipsburg, NJ, USA). Self-limiting etch was stopped once 30 × 30 μm^2 ^square windows were achieved in SiO_2_. The thickness of the SiO_2 _membranes were then reduced to 300 nm by reactive ion etching (RIE) using tetraflouromethane at 100 W and gas flow rate of 15 sccm. The etch rate of the RIE was characterized using a reflectometer (Ocean Optic, Dunedin, FL, USA). All samples were cleaned with piranha solution before FIB (Carl Zeiss, Peabody, MA, USA) drilling. The free-standing oxide membranes were drilled with the FIB to create the initial pores. The FIB process was optimized first in terms of drilling time and milling current while the acceleration voltage of 30 kV was fixed. HRTEM (Hitachi High Technologies America, Inc., Schaumburg, IL, USA) operating at 300 kV was used to image the nanopores and to characterize their diameters.

### High temperature shrinking process

The heating furnace was first turned on to raise the temperature to the desired range. All samples were put together in a horizontal carrier inside the furnace. The samples were allowed to heat up for 30 s before starting the actual processing time. The nitrogen flow rate of 20 sccm was maintained throughout the shrinking process. After the desired amount of time, the samples were taken out of furnace to cool down to room temperature. All the samples were cleaned with argon-oxygen plasma for 5 min before and after every thermal processing step to avoid hydrocarbon contamination.

## Conclusions

We demonstrated a new technique to shrink nanopores in oxide membranes with nanometer precision. The shrinking process is controlled and repeatable. In contrast to TEM or FESEM shrinking methods, our process can be used to shrink many napopore dyes in parallel. We processed 5 to 10 dyes in one run and achieved similar shrinking rates. Our technique has an additional advantage in that it did not change the chemical composition of the pore walls. The oxide layer is softened under high temperature and is allowed to diffuse due to surface diffusion of viscous oxide.

## Abbreviations

DI: de-ionized; EDS, energy dispersive X-ray spectroscopy; FESEM: field emission scanning electron microscope; FIB: focused ion beam; HRTEM: high-resolution transmission electron microscope; PR: photoresist; RIE, reactive ion etching; TEM: transmission electron microscope; TMAH: tetramethylammonium hydroxide.

## Competing interests

The authors declare that they have no competing interests.

## Authors' contributions

WA fabricated the nanopores and carried out the thermal shrinking process. WA and AI did the characterization and imaging of the nanopores. WA and SMI developed the conceptual framework and wrote the paper. SMI designed the experiments and supervised the work.
